# Metabolic Reprogramming at the Tumor–Immune Interface in Hepatocellular Carcinoma

**DOI:** 10.3390/cells15151357

**Published:** 2026-07-28

**Authors:** Weiming Zhao, Ping Li

**Affiliations:** 1 Institute of Biomedical Sciences, Henan Academy of Sciences, Zhengzhou 450046, China; 2 College of Life Science, Henan Normal University, Xinxiang 453007, China

**Keywords:** hepatocellular carcinoma, metabolic reprogramming, immune, interface

## Abstract

Hepatocellular carcinoma (HCC) arises predominantly in chronic liver disease with a uniquely tolerogenic microenvironment. Immune checkpoint inhibitors (ICIs) have improved the prognosis of advanced HCC, yet most patients exhibit low response rates or therapeutic resistance due to the highly immunosuppressive tumor microenvironment. Metabolic reprogramming is not only a core hallmark of HCC but also a key regulatory axis connecting tumor cells and the immune system. HCC cells exhibit pronounced Warburg glycolysis, upregulated glutaminolysis, aberrant lipid storage and oxidation, enhanced ketone metabolism, and altered polyamine flux. These metabolic alterations lead to nutrient competition, lactate accumulation, amino acid depletion, and oncometabolite signaling, resulting in T cell exhaustion, macrophage polarization, T cell expansion, and impaired dendritic cell function, thereby influencing tumor progression, immune escape, and therapeutic resistance. Targeting metabolic–immune crosstalk represents a promising strategy for reversing immunosuppression and enhancing the efficacy of immunotherapy. In this review, we systematically summarize the core patterns of metabolic reprogramming in HCC, dissect the molecular mechanisms of metabolic crosstalk at the tumor–immune interface, and discuss the role of immunometabolic remodeling in therapeutic resistance. This review aims to provide a comprehensive theoretical basis and new research directions for improving the efficacy of HCC treatment by targeting the metabolic–immune regulatory axis.

## 1. Introduction

Hepatocellular carcinoma (HCC) is the predominant form of primary liver cancer and remains a leading cause of cancer-related mortality worldwide [1]. Unlike many other solid malignancies, HCC develops in an organ physiologically specialized for immune tolerance. Constant exposure of the liver to dietary antigens, microbial products, and metabolic intermediates delivered through the portal circulation necessitates tight immune regulation, which is maintained by regulatory T cells (Tregs), tolerogenic dendritic cells (DCs), Kupffer cells, and immunosuppressive cytokines such as IL-10 and TGF-β [2,3]. Although essential for preventing excessive inflammation, this tolerogenic baseline may also facilitate malignant transformation and immune escape.

Immune checkpoint inhibitors (ICIs), particularly those targeting PD-1/PD-L1 and CTLA-4, have improved outcomes for selected patients with advanced HCC [4,5]. However, objective response rates remain modest, and both primary and acquired resistance are common. These observations indicate that immune checkpoint signaling alone does not fully explain therapeutic failure. Instead, resistance frequently emerges from broader interactions among tumor cells, stromal compartments, and immune populations within the tumor microenvironment (TME).

Among these regulatory layers, metabolic reprogramming has emerged as a central determinant of HCC progression and immune dysfunction. Because the liver is a major metabolic organ, malignant transformation profoundly alters pathways governing glucose utilization, amino acid turnover, lipid handling, bile acid signaling, and mitochondrial homeostasis. These changes not only support tumor proliferation and stress adaptation but also reshape immune-cell behavior through nutrient depletion, accumulation of suppressive metabolites, redox imbalance, and epigenetic remodeling.

Therefore, metabolism should be viewed not merely as a tumor-intrinsic hallmark, but as a regulatory hub at the tumor–immune interface in HCC. Understanding how metabolic rewiring coordinates immune suppression may provide new opportunities for patient stratification and rational combination therapies. In this review, we summarize major metabolic programs in HCC, discuss how they influence antitumor immunity, evaluate their relevance to therapeutic resistance, and highlight key challenges for clinical translation.

## 2. Metabolic Reprogramming in HCC

Metabolic rewiring in HCC does not occur through isolated pathways. Rather, glycolysis, glutamine utilization, lipid remodeling, ketone metabolism, bile acid signaling, and polyamine turnover form an interconnected network that supports tumor growth, survival under stress, and immune escape. Although these pathways are discussed separately below for clarity, substantial functional overlap exists among them.

### 2.1. Glycolysis

Enhanced aerobic glycolysis is one of the most reproducible metabolic features of HCC. Even under oxygen-replete conditions, tumor cells preferentially utilize glycolysis to generate ATP and biosynthetic intermediates, thereby sustaining rapid proliferation. Multiple oncogenic programs—including PI3K/AKT, MYC, and HIF-1α signaling—converge to increase expression of glucose transporters and glycolytic enzymes such as GLUT1, HK2, PKM2, and LDHA [6,7,8,9,10]. Beyond energy production, glycolysis contributes to redox homeostasis and anabolic metabolism through coupling with the pentose phosphate pathway and nucleotide synthesis. Lactate accumulation further stabilizes HIF-1α and promotes angiogenic and immunosuppressive signaling, creating a self-reinforcing loop. Collectively, current evidence suggests that glycolysis is not only a metabolic phenotype but also a dominant adaptive program linking proliferation, hypoxia tolerance, and immune evasion in HCC.

### 2.2. Glutamine

Many HCC tumors exhibit increased dependence on glutamine as a carbon and nitrogen source. Glutamine fuels the tricarboxylic acid cycle, supports nucleotide biosynthesis, and maintains glutathione-mediated redox buffering. Upregulation of glutaminase (GLS1), altered splicing programs, and MYC-related signaling all contribute to enhanced glutaminolysis [11,12,13,14,15]. Functionally, glutamine metabolism appears particularly important under metabolic stress, where it compensates for fluctuating glucose availability. In addition, glutamine-derived intermediates feed into the hexosamine biosynthetic pathway, linking nutrient sensing to protein O-GlcNAcylation and transcriptional control. Although GLS inhibitors have shown preclinical promise, monotherapy responses remain inconsistent, suggesting that glutamine dependence may vary across HCC subtypes and treatment contexts.

### 2.3. Fatty Acid

Lipid remodeling is increasingly recognized as a major metabolic axis in HCC, especially in the context of metabolic syndrome and steatotic liver disease. Tumor cells frequently upregulate de novo lipogenesis through FASN, SCD1, and SREBP1 signaling, thereby supporting membrane synthesis, signaling lipid production, and resistance to oxidative stress [16,17,18,19,20]. At the same time, fatty acid oxidation and lipophagy may provide metabolic flexibility during nutrient limitation. These dual programs indicate that HCC cells can switch between lipid storage, synthesis, and utilization according to environmental pressure. Importantly, lipid metabolism is also closely linked to ferroptosis sensitivity, suggesting therapeutic opportunities. However, which lipid pathway is dominant likely depends on etiology, tumor stage, and molecular subtype.

### 2.4. Ketone

Ketone metabolism in HCC appears context-dependent. Some tumors upregulate ketolytic enzymes such as OXCT1 to exploit ketone bodies as an alternative fuel source, whereas impaired ketogenesis has been associated with glycolytic compensation and resistance to systemic therapy [21,22,23,24]. These findings suggest that ketone pathways may reflect metabolic plasticity rather than a universal hallmark of HCC. Their translational relevance remains promising but comparatively less established than glycolysis or lipid metabolism.

### 2.5. Bile Acid

Because bile acid synthesis and recycling are liver-specific functions, dysregulated bile acid metabolism represents a distinctive feature of HCC. Altered bile acid composition can influence inflammation, fibrosis, microbiota structure, and receptor signaling through the FXR and TGR5 pathways [25,26]. Emerging studies further indicate that bile acids participate in gut–liver immune communication by modulating chemokine expression and immune-cell recruitment. Although mechanistic understanding remains incomplete, bile acid signaling may represent a uniquely liver-centered therapeutic axis.

### 2.6. Polyamine

Polyamine biosynthesis and turnover are frequently elevated in HCC and are associated with proliferation, stress tolerance, and aggressive clinical behavior [27,28,29]. In addition to supporting tumor cell growth, secreted polyamine metabolites may remodel the immune microenvironment by promoting suppressive myeloid responses and Treg recruitment. Compared with other pathways, polyamine metabolism has received less translational attention but may represent a convergent immunosuppressive hub worthy of further investigation.

**Figure 1:**
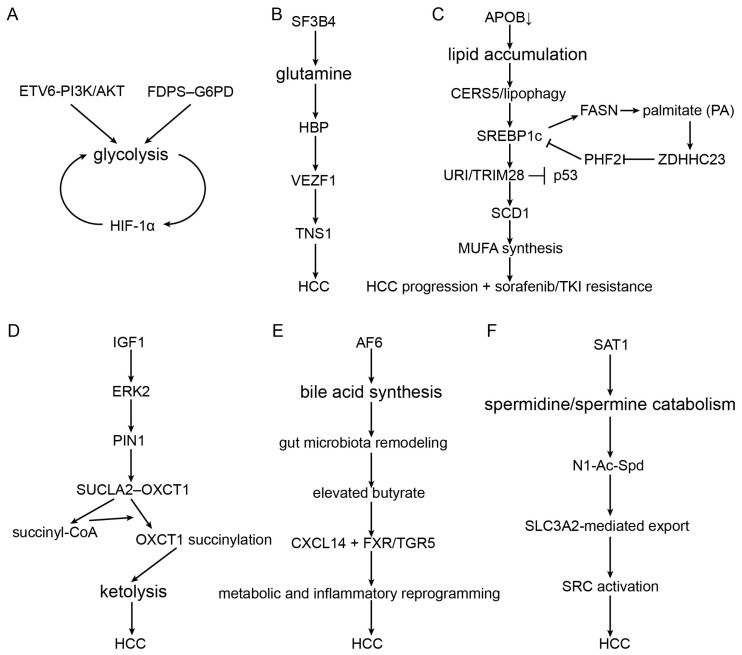
Metabolic reprogramming in HCC. (**A**) Glycolysis is activated by ETV6-PI3K/AKT and FDPS-G6PD signaling, forming a positive feedback loop with HIF-1α. (**B**) SF3B4 drives glutamine flux into the hexosamine biosynthetic pathway (HBP), promoting HCC progression via the VEZF1-TNS1 axis. (**C**) Lipid accumulation and lipophagy drive SREBP1c-mediated MUFA synthesis via URI/TRIM28 inhibition of p53, supporting HCC progression and sorafenib/TKI resistance. (**D**) The IGF1-ERK2-PIN1 axis promotes SUCLA2-OXCT1-driven ketolysis via succinylation, fueling HCC growth. (**E**) AF6-mediated bile acid synthesis remodels the gut microbiota, elevating butyrate and reprogramming immunity via CXCL14 and FXR/TGR5 signaling. (**F**) SAT1-mediated polyamine catabolism produces N^1^-Ac-Spd, which is exported via SLC3A2 to activate SRC signaling and drive HCC.

Taken together, metabolic reprogramming in HCC is best viewed as a dynamic and interconnected network rather than a collection of isolated pathways. Glycolysis and lipid metabolism currently have the strongest translational evidence, whereas bile acid, ketone, and polyamine pathways may offer liver-specific or niche-dependent vulnerabilities that require further validation (Figure 1).

## 3. Metabolic Crosstalk at the Tumor–Immune Interface in HCC

Metabolic crosstalk at the tumor–immune interface can be conceptually divided into three overlapping modes: (1) nutrient competition, in which tumor cells deprive immune cells of essential substrates; (2) metabolite signaling, whereby tumor-derived metabolites directly reprogram immune-cell fate; and (3) epigenetic remodeling driven by metabolic intermediates. These processes collectively shape immune dysfunction and therapeutic resistance in HCC.

### 3.1. Metabolic Heterogeneity of Immune Cells in the Tumor Microenvironment

Immune cells within the HCC microenvironment are not metabolically uniform. Instead, their functional states are tightly linked to nutrient availability and intracellular metabolic programs. Effector CD8^+^ T cells rely heavily on glycolysis to sustain proliferation and cytotoxicity, whereas memory T cells preferentially utilize fatty acid oxidation and oxidative phosphorylation. Exhausted T cells display impaired mitochondrial fitness and reduced metabolic flexibility, limiting responsiveness to ICIs [30,31,32,33,34,35,36,37].

Regulatory T cells adapt to nutrient shortage via fatty acid oxidation and glutaminolysis to maintain suppression. Pro-inflammatory macrophages and dendritic cells rely on glycolysis, whereas tolerogenic/M2-like subsets favor lipid metabolism and mitochondrial respiration [38,39].

**Figure 2:**
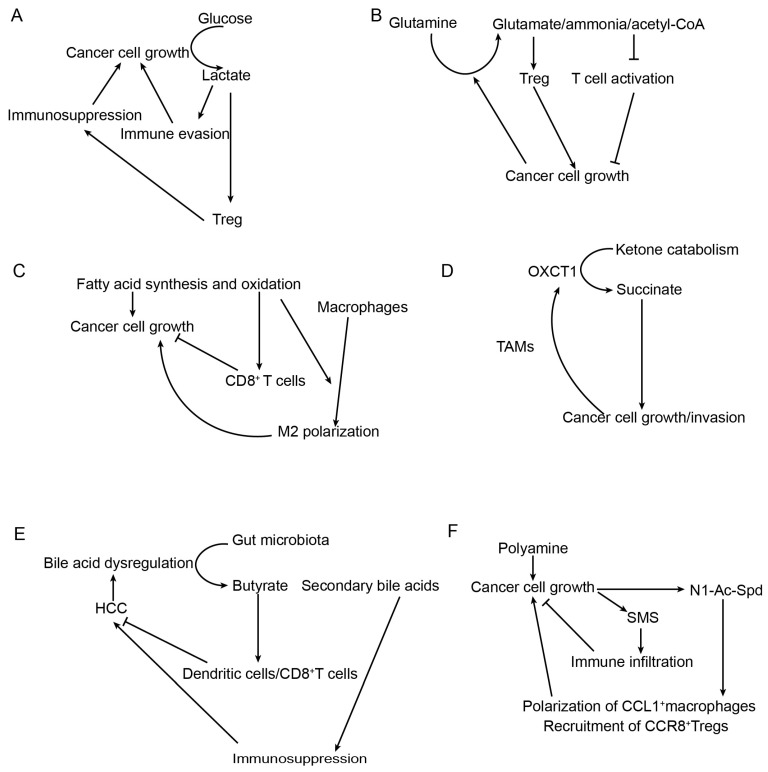
Metabolic crosstalk at the tumor–immune interface in HCC.(**A**) Aerobic glycolysis produces lactate, promoting immune evasion and Treg expansion. (**B**) Glutamine metabolism generates glutamate, ammonia, and acetyl-CoA, which support tumor growth while inhibiting T cell activation and favoring Treg function. (**C**) Dysregulated fatty acid synthesis and oxidation impair CD8^+^ T cell function and drive M2-like polarization of macrophages. (**D**) Ketone catabolism via OXCT1 generates succinate, promoting cancer cell growth/invasion and shaping protumorigenic tumor-associated macrophages (TAMs). (**E**) Bile acid dysregulation interacts with the gut microbiota to modulate immunity: secondary bile acids promote immunosuppression, while microbiota-derived butyrate enhances dendritic cell and CD8^+^ T cell antitumor responses. (**F**) Hyperactivated polyamine metabolism produces N^1^-acetylspermidine (N^1^-Ac-Spd), driving polarization of CCL1^+^ macrophages and recruitment of CCR8^+^ regulatory T cells, thereby blunting antitumor immunity.

Other immune cells are also metabolically impaired: lactate, NAD^+^ depletion, and mitochondrial stress suppress NK cell cytotoxicity; lactate polarizes neutrophils toward a protumor PD-L1^+^ phenotype; MDSCs enhance glycolysis and inhibit T/NK cells via ARG1 and iNOS. Galectin-4-mediated glycolytic signaling reduces CD8^+^ T cell infiltration and confers resistance to immunotherapy. Together, nutrient competition and immune metabolic heterogeneity form a network that drives immune escape, tumor progression, and therapy resistance in HCC [40,41,42] (Figure 2).

### 3.2. Glycolysis: From Energetic Adaptation to Immunometabolic Checkpoint

Among all metabolic pathways, glycolysis likely exerts the most immediate immunological impact in HCC. High glucose consumption by tumor cells restricts nutrient availability for CD8^+^ T cells and NK cells, thereby impairing proliferation, cytokine production, and cytotoxicity. In parallel, lactate accumulation promotes extracellular acidification, enhances Treg function, and supports macrophage polarization toward suppressive phenotypes [43,44,45,46,47]. Several upstream pathways regulate this phenotype, but current evidence converges on two dominant downstream mechanisms: glucose deprivation and lactate signaling. This distinction is clinically relevant, as targeting terminal outputs such as lactate transport or acidity may prove more effective than inhibiting individual upstream oncogenic drivers. Thus, glycolysis should be regarded as an immunometabolic checkpoint that couples tumor bioenergetics with immune escape.

### 3.3. Glutaminolysis–Ammonia Axis: Remodeling Nitrogen Metabolism and Immune Balance

HCC cells highly express glutamine transporters (SLC38A1, SLC38A2, SLC1A5) to avidly take up glutamine from the TME. Glutamine is catabolized by GLS into glutamate and ammonia; ammonia was once considered a toxic waste product but is now recognized as a Treg-selective metabolic signal. Accumulated acetyl-CoA activates VDAC1 transcription via H3K27ac, promoting mitophagy and HBV-related HCC [48]. Additionally, acetyl-CoA supports ACAT1-mediated conversion of GABA to 4-Ac-GABA, which inhibits the AKT1 pathway in CD8^+^ T cells, thereby suppressing T cell activation, infiltration, and antitumor immunity [49].

### 3.4. Fatty Acid and Lipid Metabolism: Linking Metabolic Syndrome to Immune Dysfunction

Dysregulation of fatty acid and lipid metabolism is a hallmark of HCC and acts as a double-edged sword in immunometabolism, driving both tumor progression and immune remodeling. FASN is overexpressed to enhance de novo lipogenesis, supporting proliferation, invasion, poor prognosis, and therapy resistance [50]. FAO (mediated by CPT1A/CPT2) is also activated to sustain energy supply under nutrient stress [51,52].

In the tumor–immune microenvironment, omega-3 PUFAs enhance antitumor immunity by boosting CD8^+^ T cell and NK cell function [53,54,55]. Conversely, tumor-derived unsaturated fatty acids are transferred to TAMs via FABP5, activating PPARγ and inducing immunosuppressive phenotypes [56]. Cholesterol accumulation also impairs CD8^+^ T cell function and promotes M2-like macrophage polarization [57]. Thus, lipid metabolism in HCC exerts context-dependent immunomodulatory effects shaped by lipid species, cell status, and TME composition.

### 3.5. Ketone Metabolism: Succinate as an Oncometabolite and Epigenetic Regulator

Ketone body metabolism is an emerging metabolic pathway that is abnormally regulated in HCC, and succinate, a key intermediate of ketone body metabolism, acts as an oncometabolite and epigenetic regulator, participating in the progression of HCC and immune escape. TAMs exhibit abnormal ketone body metabolism, which is mediated by the high expression of 3-oxoacyl-CoA transferase 1 (OXCT1), a key enzyme of ketone body catabolism. OXCT1 is highly expressed in TAMs of HCC tissues. OXCT1-high TAMs accumulate succinate, a byproduct of ketone body catabolism. First, succinate promotes the proliferation and invasion of HCC cells by activating the hypoxia-inducible factor 1α (HIF-1α) signaling pathway [58]. In addition, succinate inhibits α-KG-dependent demethylases (KDM5 family), leading to H3K4me3 enrichment at the ARG1 promoter. Arginase 1 (ARG1) upregulation depletes microenvironmental L-arginine, impairing CD8+ T cell proliferation and effector function [59].

### 3.6. Bile Acid Metabolism: Gut–Liver Axis and Immune Recruitment

As the central organ for bile acid synthesis and recycling, the liver makes bile acids key immunometabolic mediators in HCC [60]. Aberrant bile acid metabolism (e.g., GCDCA accumulation) promotes a fibrotic TME and tumor progression by activating S1PR2 and inducing protumorigenic cancer-associated fibroblasts [61].

The gut–liver axis also shapes HCC via the microbiota and bile acid crosstalk [62]. Loss of AF6 elevates primary bile acids, remodels the microbiota, and increases butyrate, which upregulates CXCL14 to recruit dendritic cells and enhance CD8^+^ T cell immunity [25,63]. Conversely, dysregulated secondary bile acids and chronic LXRα activation trigger oxysterol accumulation, activate IL-6/STAT3 signaling, and induce MDSC-mediated immunosuppression [64]. Bile acid metabolism thus serves as a liver-specific metabolic–immune bridge linking local tumor behavior to systemic microbial signals.

### 3.7. Polyamine Metabolism: A Convergent Immunosuppressive Hub

In HCC, polyamine metabolism is hyperactivated via upregulation of ODC1 and SAT1 [65,66], promoting tumor proliferation, metastasis, and immunosuppression. A polyamine-based PA score effectively predicts HCC prognosis and immunotherapy response [67].

Mechanistically, inflammatory macrophages induce SAT1 in tumor cells, triggering production and extracellular release of N1-acetylspermidine via SLC3A2. This metabolite activates SRC signaling, driving CCL1^+^ macrophage polarization and CCR8^+^ Treg recruitment to suppress antitumor immunity [28]. Additionally, high SMS expression upregulates immune checkpoints and impairs immune pathways, further reinforcing an immunosuppressive microenvironment and blunting ICI efficacy [68].

For clarity, the key metabolic pathways, their drivers, metabolites, and corresponding immune effects are summarized in Table 1.

## 4. Immunometabolic Remodeling and Therapeutic Resistance

### 4.1. Metabolic Determinants of ICI Response

Although ICIs have improved the treatment landscape of advanced HCC, durable responses are limited to a subset of patients. Increasing evidence suggests that responsiveness is shaped not only by immune checkpoint expression, but also by the metabolic architecture of the tumor microenvironment (TME) [69,70,71].

Tumors enriched for glycolysis often display glucose depletion, lactate accumulation, hypoxia, and exclusion of effector lymphocytes, all of which are associated with poor ICI sensitivity. In contrast, tumors retaining greater oxidative metabolism or metabolic differentiation may exhibit a more permissive immune milieu. Spatial transcriptomic studies further suggest that clinical responders undergo coordinated remodeling of both immune-cell localization and tumor metabolism, rather than immune activation alone [72]. These findings support an important conceptual shift: metabolic state may function as both a biomarker and a therapeutic determinant of ICI response. However, prospective validation remains limited, and no metabolism-based classifier has yet entered routine clinical practice.

### 4.2. Metabolite-Mediated T Cell Exhaustion, Macrophage M2 Polarization, and Dendritic Cell Dysfunction

Tumor-derived metabolites can directly impair antitumor immunity. Lipid intermediates, lactate, methionine-cycle metabolites, and cholesterol derivatives have all been implicated in promoting CD8^+^ T cell exhaustion, reducing immune-cell trafficking, or limiting effector cytokine production [73,74,75,76,77].

Macrophages are similarly susceptible to metabolic reprogramming. Lactate, oxysterols, and fatty acids can drive polarization toward suppressive M2-like phenotypes, reinforcing immune evasion and therapeutic resistance [78,79,80]. Dendritic cells may also lose antigen-presenting capacity under chronic metabolic stress, reducing priming of tumor-reactive T cells [81,82,83,84,85]. Importantly, many of these signals converge phenotypically despite diverse upstream mechanisms. Thus, reversing immune dysfunction may not require targeting every metabolite individually, but rather restoring broader immune metabolic fitness.

### 4.3. Hypoxia and Immune Suppression

Hypoxia is a central integrator of metabolic and immune suppression in HCC. Stabilization of HIF-1α promotes glycolysis, lactate production, angiogenesis, extracellular matrix remodeling, and checkpoint signaling, thereby creating multiple barriers to effective immunity [86,87,88,89,90]. Because hypoxia simultaneously affects tumor cells, vasculature, stromal compartments, and immune populations, it may represent a higher-order therapeutic target than isolated metabolic enzymes. Nevertheless, safe and durable hypoxia-directed strategies remain challenging, and clinical efficacy in HCC is not yet established.

Taken together, therapeutic resistance in HCC is rarely explained by a single pathway. Instead, metabolic dysfunction, hypoxia, immune exhaustion, and stromal remodeling cooperate to generate a treatment-refractory ecosystem. This complexity helps explain why many single-agent metabolic therapies have shown limited benefit.

## 5. Targeting Tumor–Immune Metabolic Interactions in HCC

Given the multifactorial nature of immunosuppression, therapeutic targeting of metabolism in HCC should be viewed as a means to recondition the TME rather than simply starve tumor cells. Current strategies mainly focus on glycolysis, amino acid metabolism, lipid pathways, and engineered immune-cell fitness.

### 5.1. Glycolysis Inhibitors

Inhibitors of glucose transport, glycolytic enzymes, and lactate transport have shown antitumor activity in preclinical HCC models [91,92,93,94,95,96]. Mechanistically, these agents may reduce tumor proliferation while simultaneously relieving nutrient competition and lactate-mediated immune suppression. However, glycolysis is also essential for activated immune cells and normal tissues, raising concerns regarding therapeutic index. As a result, systemic glycolysis blockade may be less practical than selective targeting of downstream outputs such as lactate export, tumor acidity, or hypoxia-associated signaling.

### 5.2. IDO/Tryptophan Axis Targeting

Indoleamine 2,3-dioxygenase (IDO) is highly expressed in HCC and promotes immune evasion by depleting tryptophan and accumulating kynurenine, thereby expanding regulatory T cells and impairing effector T cell function. Preclinical studies show that IDO also mediates adaptive resistance to ICIs, as anti-CTLA-4 and anti-PD-1 therapies induce IDO through IFN-γ signaling, whereas IDO inhibition restores antitumor immunity [97].

Accordingly, several IDO-targeting strategies have been developed. Nanodelivery systems such as NLG919/PI-FVIOs improve tumor-specific release of the IDO inhibitor NLG919 and enhance magnetothermal therapy-induced immunogenic cell death, increasing cytotoxic T cell infiltration while reducing Treg expansion [98]. Prodrug nanoparticles (FU-SS-IND NPs) combine 5-FU with an IDO inhibitor to overcome chemotherapy resistance and increase PD-L1 expression, supporting combination with immune checkpoint blockade [99]. In addition, quetiapine suppresses IDO via the ERK/NF-κB pathway while inducing apoptosis and inhibiting angiogenesis and invasion [100].

Collectively, IDO inhibition remodels the immunosuppressive microenvironment and may synergize with chemotherapy, immunotherapy, and immunogenic cell death-based strategies, representing a promising therapeutic avenue for HCC.

### 5.3. Lipid Metabolism Modulators

Therapeutic targeting of lipid metabolism can simultaneously suppress tumor growth and enhance antitumor immunity in HCC. Current strategies mainly include inhibitors of fatty acid synthesis, fatty acid oxidation, and cholesterol metabolism.

Fatty acid synthesis inhibitors primarily target FASN, which is frequently upregulated in HCC. Orlistat inhibits FASN, reducing unsaturated fatty acid secretion and decreasing FABP5^+^ lipid-laden TAM infiltration, thereby relieving lipid-driven immunosuppression [101]. Similarly, TVB-3166 suppresses tumor growth in preclinical models and has entered clinical evaluation, highlighting the translational potential of blocking de novo lipogenesis [102].

Fatty acid oxidation inhibitors mainly target CPT1A, a rate-limiting enzyme in mitochondrial β-oxidation. Etomoxir promotes TAM repolarization from an immunosuppressive M2 phenotype toward a pro-inflammatory M1 state and induces tumor cell apoptosis [103]. Likewise, perhexiline enhances the efficacy of ICIs in HCC models, suggesting that FAO inhibition can strengthen antitumor immunity [104].

Cholesterol metabolism regulators also modulate immune responses. Atorvastatin reduces cholesterol accumulation in TAMs, reverses M2 polarization, and enhances antigen presentation [105]. Ezetimibe limits cholesterol uptake in tumor and immune cells, thereby improving immune activation and antitumor efficacy [106].

In addition, targeting the FABP5/PPARγ axis represents an emerging strategy linking lipid metabolism with immune regulation. FABP5 inhibition reduces lipid-laden TAM infiltration and promotes CD8^+^ T cell responses, whereas PPARγ inhibition suppresses MDSC recruitment, restores CD8^+^ T cell function, and sensitizes HCC to immune checkpoint blockade [107,108].

### 5.4. Enhancing CAR-T and Adoptive Therapy

Metabolic reprogramming of CAR-T cells can improve their survival and antitumor activity in HCC. In the immunosuppressive tumor microenvironment, CAR-T cells often develop metabolic exhaustion, which limits therapeutic efficacy [109]. Enhancing glycolysis and anti-apoptotic capacity through overexpression of glucose transporter 1 (GLUT1) or acylglycerol kinase (AGK), via PI3K/Akt signaling, increases the in vivo persistence and antitumor efficacy of GPC3-targeted CAR-T cells in HCC [110].

More broadly, adoptive cell therapy (ACT), including tumor-infiltrating lymphocytes (TILs), cytokine-induced killer (CIK) cells, and CAR-T cells, provides personalized and tumor-specific immunity by infusing autologous immune cells expanded or genetically engineered ex vivo. These approaches help overcome immune tolerance and eliminate circulating tumor cells or residual disease, offering sustained therapeutic benefit in HCC [111].

Overall, the most realistic near-term role of metabolic therapy may be as an adjunct to immunotherapy rather than a standalone treatment. Future success will likely depend on selective targeting, rational combinations, and patient stratification based on dominant metabolic dependencies.

## 6. Combined Therapeutic Strategies

### 6.1. Metabolic Inhibitors Combined with ICIs in Clinic

Several early-phase studies have explored combining metabolic modulation with ICIs in advanced HCC. For example, IDO inhibition plus nivolumab demonstrated acceptable tolerability in a small phase I cohort, but efficacy signals were modest [112].Other approaches, including DGK inhibition, remain under active investigation.

At the same time, negative studies are equally informative. The failure of pravastatin plus sorafenib and retrospective concerns regarding metformin in selected immunotherapy settings illustrate that not all metabolic interventions are beneficial [113,114]. These findings reinforce an important principle: metabolic drugs cannot be assumed to synergize with ICIs simply because they affect metabolism.

### 6.2. Novel Combined Strategies

Combination therapies that integrate local tumor control and systemic immune activation effectively enhance antitumor immunity and improve HCC outcomes. Cryoablation plus PD-1 blockade releases tumor antigens and boosts T cell activity, significantly improving response rates and survival with manageable toxicity [115]. Oncolytic virus VG161 exerts direct oncolysis and reprograms the TME toward immune activation, triggering systemic antitumor responses [116].

The LEN-TAP regimen (lenvatinib + TACE + PD-1 inhibition) achieves high conversion rates for unresectable HCC and prolongs survival. Immune biomarkers such as circulating HLA-DR^+^CD38^+^CD8^+^ T cells help stratify patients but require combined interpretation with tumor-infiltrating lymphocytes and imaging [117]. The STRIDE regimen (tremelimumab + durvalumab) also delivers durable immune activation and superior survival [118].

Although these combinations show strong promise, translating immunometabolic therapies into sustained clinical benefit remains challenging, requiring deeper mechanistic research and better patient stratification. Collectively, these findings highlight the potential of combinatorial approaches that integrate metabolic regulation and immunotherapy. A summary of current therapeutic strategies targeting HCC metabolism and immune crosstalk is provided in Table 2.

### 6.3. Future Combination Logic

Rather than combining therapies empirically, future regimens should be designed according to metabolic context. Examples may include:

Glycolytic/hypoxic tumors → PD-1 blockade + lactate/hypoxia targeting;

Lipid-rich MASLD-HCC → ICI + lipid pathway modulation;

Immune-excluded tumors → locoregional therapy + metabolic reconditioning;

T-cell-dysfunctional tumors → ACT/CAR-T + nutrient support strategies.

This biomarker-driven framework is likely to outperform one-size-fits-all combination models.

Current evidence suggests that combination therapy is more promising than metabolic monotherapy, but enthusiasm should be balanced by the limited scale of available clinical data. The next generation of trials should prioritize biological selection, adaptive design, and mechanistic endpoints.

## 7. Emerging Technologies to Decode Immunometabolism

A major barrier in HCC immunometabolism has been the inability of conventional bulk analyses to resolve spatial, cellular, and temporal heterogeneity. Emerging multi-omics technologies are now transforming the field by enabling high-resolution mapping of metabolic states together with immune architecture.

### 7.1. Single-Cell Transcriptomics

Utilizing single-cell transcriptomics, Chang et al. identify HLA-DR^+^ tumor cells as a hallmark of HBV-associated HCC, revealing their role in shaping an immunosuppressive microenvironment through immune checkpoint activation (PD-L1) and induction of CD8^+^ T cell exhaustion, thereby providing a potential predictive biomarker for immunotherapy response [119]. And Aldo et al. uncovered lipid metabolic reprogramming in Tregs and CAFs within SLD-HCC. Their ligand–receptor analysis pinpointed the TNFSF14-TNFRSF14 axis as central to establishing an immunosuppressive microenvironment marked by CD8+ T cell depletion and Treg accumulation [120]. These findings underscore a promising therapeutic strategy for HCC, grounded in the integrated understanding of its metabolic, immunological, and spatial dysregulation.

### 7.2. Spatial Omics

By integrating spatial and single-cell transcriptomics, a study uncovered the eIF3f-ACSL4 axis as a driver of fatty acid biosynthesis in HCC, revealing that the resultant lipid-rich tumor microenvironment spatially restricts CD8^+^ T cell infiltration, thereby linking metabolic reprogramming to immune evasion and identifying eIF3f as a potential dual-action therapeutic target [121]. Using a spatial multi-omics platform, another study defined a unique tumor–immune–CAF “interface” zone in HCC, revealing that the enhanced metabolic crosstalk in this region is characterized by the upregulation of lactic acid and long-chain polyunsaturated fatty acids. Furthermore, it demonstrated that CAF-derived lactic acid drives macrophage M2 polarization, thereby elucidating how spatially organized metabolic–immune crosstalk shapes an immunosuppressive microenvironment [122]. These findings provide new insights for developing combination therapies targeting metabolic pathways.

### 7.3. Metabolomics

Through integrated metabolomic and lipidomic analyses, a study established a fatty acid degradation (FAD) pathway-based molecular classification, identifying three HCC subtypes (F1-F3) with distinct metabolic and immune features. F1 subtype: Low FAD activity, marked lipid accumulation, and an immunosuppressive microenvironment (high Tregs, exhausted CD8^+^ T cells, M2 macrophages). This subtype relies on lipogenesis rather than fatty acid breakdown, leading to lipid-laden immunosuppressive macrophages and T cell dysfunction, and is sensitive to anti-PD-1/PD-L1 immunotherapy and sorafenib. F2 subtype: Intermediate FAD activity and mixed immune metabolic features, with moderate therapeutic responsiveness. F3 subtype: High FAD activity, enhanced mitochondrial fatty acid oxidation, and a relatively immune-active microenvironment; this subtype shows better response to transarterial chemoembolization (TACE) [123]. Circulating metabolites, lipids, and related signatures also offer promise as minimally invasive biomarkers for treatment monitoring. However, systemic metabolic signals can be influenced by liver function, diet, microbiota composition, and comorbid disease, which may reduce specificity. Thus, metabolomics is highly promising but will likely be most informative when integrated with clinical and molecular data rather than used alone.

Together, emerging technologies are shifting the field from descriptive metabolism analyses toward precision immunometabolism. The key next step is not generating more data, but translating multi-omic complexity into clinically actionable biomarkers and therapeutic decisions.

## 8. Challenges

Despite rapid progress, several major barriers continue to limit the clinical translation of immunometabolic therapies in HCC.

### 8.1. Metabolic Heterogeneity

HCC is not a metabolically uniform disease. Tumors differ by etiology, genomic drivers, differentiation state, vascularization, and prior treatment exposure. Even within individual lesions, multiple metabolic states may coexist [124]. This heterogeneity likely explains why therapies targeting a single pathway often produce inconsistent outcomes.

### 8.2. Narrow Therapeutic Window

Many candidate targets, particularly glycolysis and mitochondrial metabolism, are also essential in normal hepatocytes and activated immune cells. As a result, systemic inhibition may cause hepatotoxicity, fatigue, metabolic disturbance, or unintended immune suppression [125,126,127,128,129].

### 8.3. Dynamic Adaptation and Resistance

Tumors can compensate for inhibition of one pathway by switching to alternative nutrient sources or engaging stromal support programs. This metabolic plasticity suggests that static treatment models may be inadequate.

### 8.4. Biomarker Deficiency

No validated biomarker currently identifies which HCC patients are most likely to benefit from metabolic therapy. This remains a central obstacle for trial design and personalized treatment [130].

### 8.5. Etiology-Specific Complexity

Viral hepatitis-related HCC, MASLD-associated HCC, and alcohol-related HCC arise in distinct inflammatory and metabolic environments. Pooling these populations may obscure therapeutic signals.

Overall, the major challenge is not proving that metabolism matters—it clearly does—but determining when, where, and in whom specific metabolic interventions are clinically meaningful.

## 9. Future Perspectives

Despite these challenges, the field of HCC immunometabolism holds significant promise for therapeutic innovation. The convergence of emerging multi-disciplinary technologies—including single-cell transcriptomics, spatial omics, and metabolomics—is transforming our understanding of the tumor–immune metabolic landscape.

First, precision subtyping based on integrated metabolic and immune profiling will become a cornerstone of future HCC treatment [131]. By leveraging multi-omics platforms, patients can be stratified into distinct subtypes according to their metabolic phenotypes and immune microenvironment characteristics. For example, tumors exhibiting enhanced glycolysis may be vulnerable to glycolysis inhibitors combined with ICIs, while those with dysregulated lipid metabolism may benefit from lipid metabolism modulators combined with ICIs. Liquid biopsy-based metabolomics enables non-invasive tracking of metabolic evolution during treatment, facilitating timely regimen adjustments.

Second, the development of highly specific metabolic inhibitors and advanced targeted delivery systems offers a viable strategy to mitigate systemic toxicity [132]. Next-generation inhibitors are being designed to exploit tumor-specific dependencies, targeting enzymes such as HK2 and FASN that are overexpressed in HCC but low in normal tissues [133,134]. Nanotechnology-based platforms—including tumor-targeted lipid nanoparticles and nanogels—enable preferential drug accumulation in the tumor microenvironment, improving therapeutic index and enabling synchronized modulation of the tumor–immune metabolic axis.

Third, integrative multi-omics approaches continue to uncover novel metabolic checkpoints and therapeutic targets. Recent studies have identified OXCT1 (mediating ketone body metabolism in exhausted T cells), the bile acid/butyrate/CXCL14 axis linking gut microbiota to hepatic immune surveillance, and SLC3A2 (driving immunosuppressive macrophage polarization via N1-acetylspermidine) [59]. Mechanistic interrogation reveals synergistic potential—for instance, combining metabolic inhibitors with ferroptosis inducers enhances tumor cell death, while targeting SLC3A2 synergizes with PD-1 blockade.

Finally, the convergence of immunology, metabolomics, bioinformatics, nanotechnology, and clinical medicine will accelerate translation [122]. Spatial transcriptomics combined with metabolomics maps metabolic niches supporting immune evasion; bioinformatics algorithms predict patient-specific vulnerabilities; nanotechnology enables precision modulation; and clinical cohorts provide real-world validation.

In conclusion, metabolic reprogramming at the tumor–immune interface plays a key role in HCC progression and therapy resistance. Although significant challenges remain, the rapid advancement of emerging technologies and deepening understanding of immunometabolism position the field for transformative progress, bringing new hope to HCC patients.

## 10. Summary and Conclusions

Metabolic reprogramming is a defining feature of HCC and a major regulator of tumor–immune interactions. Alterations in glycolysis, amino acid utilization, lipid metabolism, ketone pathways, bile acid signaling, and polyamine turnover collectively shape nutrient availability, metabolite signaling, and immune-cell fitness within the tumor microenvironment.

Current evidence indicates that metabolism contributes not only to tumor growth, but also to immune evasion, resistance to ICIs, and variable responses to combination therapy. However, these pathways are neither uniform across patients nor equally dominant across disease stages. This heterogeneity likely underlies the limited success of many single-agent metabolic interventions.

Importantly, the field is now entering a more mature phase. Emerging single-cell, spatial, and metabolomic technologies are enabling biologically informed patient stratification, while newer therapeutic approaches seek to selectively recondition the tumor microenvironment rather than indiscriminately inhibiting core metabolism.

Several limitations remain, including incomplete understanding of temporal metabolic evolution, insufficient attention to disease etiology, lack of validated biomarkers, and the scarcity of large prospective clinical studies. Nonetheless, these challenges define a roadmap rather than a dead end.

In conclusion, metabolism should no longer be viewed as a background feature of HCC biology, but as a clinically relevant layer of tumor–immune regulation. Harnessing this insight may open new avenues toward more precise and durable therapies for patients with HCC.

## Figures and Tables

**Table 1:** Summary of metabolic pathways and their immune effects in HCC.

Metabolic Pathway	Key Drivers/Enzymes	Key Metabolites	Major Immune Effects
Glycolysis	GLUT1, HK2, LDHA, HIF-1α	Lactate	Inhibits CD8^+^ T/NK cells; induces Treg activation; promotes M2 macrophage polarization; upregulates PD-L1
Glutamine metabolism	GLS1, SLC38A1/2, GFAT1	Glutamate, ammonia, 4-Ac-GABA	Deprives T cells of nutrients; suppresses CD8^+^ T activation; promotes Treg differentiation
Fatty acid/lipid metabolism	FASN, SCD1, CPT1A, FABP5	Unsaturated fatty acids, cholesterol	Lipid overload in TAMs; impairs CD8^+^ T function; drives M2 polarization; inhibits ferroptosis
Ketone metabolism	OXCT1, SUCLA2	Succinate	Stabilizes HIF-1α; upregulates ARG1; depletes arginine; suppresses CD8^+^ T cytotoxicity
Bile acid metabolism	AF6, FXR, TGR5	Primary/secondary bile acids, butyrate	Modulates DC recruitment; reshapes gut microbiota; regulates CXCL14-mediated immune infiltration
Polyamine metabolism	ODC1, SAT1, SLC3A2, SMS	N^1^-Ac-Spd	Activates SRC; induces CCL1^+^ macrophages; recruits CCR8^+^ Tregs; blunts ICI efficacy

**Table 2:** Current therapeutic strategies targeting HCC metabolism.

Metabolic Pathway	Target/Drug	Mechanism	Stage	Key Effect
Glycolysis	2-DG, 3-BP	HK2 inhibition, glycolysis blockade	Preclinical	Induce apoptosis, reduce tumor growth
Glycolysis	GSK2837808A, FX11	LDHA inhibition	Preclinical	Reduce lactate, relieve immunosuppression
Glycolysis	AZD3965	MCT1 inhibitor	Clinical/Preclinical	Block lactate transport, inhibit M2 polarization
Glycolysis	WZB117, STF-31	GLUT1 inhibitors	Preclinical	Reduce glucose uptake, restore CD8^+^ T cell function
Glycolysis	LGP nanogels	Glucose depletion + ROS production	Preclinical	Activate DCs and CD8^+^ T cells
Glutamine metabolism	CB-839 (Telaglenastat)	GLS1 inhibitor	Clinical/Preclinical	Increase ROS, sensitize to chemotherapy
Lipid metabolism	Orlistat	FASN inhibitor	Preclinical	Reduce lipid-mediated immunosuppression
Lipid metabolism	TVB-3166	FASN inhibitor	Clinical trial	Suppress tumor growth
Fatty acid oxidation	Etomoxir	CPT1A inhibitor	Preclinical	Promote M1 macrophage polarization
Fatty acid oxidation	Perhexiline	CPT1A inhibitor	Preclinical	Enhance ICI efficacy
Cholesterol metabolism	Atorvastatin	Cholesterol synthesis inhibitor	Clinical	Improve antigen presentation
Cholesterol metabolism	Ezetimibe	Cholesterol uptake inhibitor	Preclinical	Enhance immune activation
IDO/tryptophan axis	NLG919	IDO inhibitor	Preclinical/Clinical	Reverse T cell suppression
IDO/tryptophan axis	BMS-986205	IDO1 inhibitor	Clinical	Enhance ICI efficacy
Combination therapy	Nivolumab + IDO inhibitor	Immunometabolic combination	Clinical	Improve response rate
Combination therapy	Cryoablation + PD-1 blockade	Local + systemic immune activation	Clinical	Improve OS and PFS
Nanotherapy	C-B-M-Mn nanovesicles	STING activation + glycolysis inhibition	Preclinical	Promote DC maturation and T cell recruitment

## Data Availability

No new data were created or analyzed in this study.

## Abbreviations

The following abbreviations are used in this manuscript:

HCC	hepatocellular carcinoma
ICIs	immune checkpoint inhibitors
Tregs	regulatory T cells
DCs	dendritic cells
TME	tumor microenvironment
FDPS	farnesyl diphosphate synthase
FLC	fibrolamellar carcinoma
PA	palmitic acid
TKIs	tyrosine kinase inhibitors
FASN	fatty acid synthase
TAMs	tumor-associated macrophages
UFAs	unsaturated fatty acids
NKs	natural killer cells
CAFs	cancer-associated fibroblasts
IDO	indoleamine 2,3-dioxygenase
MDSCs	myeloid-derived suppressor cells
ACT	adoptive cell therapy
